# The Effectiveness of an Oral Fixed-Dose Combination of Netupitant and Palonosetron (NEPA) in Patients With Multiple Risk Factors for Chemotherapy-Induced Nausea and Vomiting: A Multicenter, Observational Indian Study

**DOI:** 10.7759/cureus.29094

**Published:** 2022-09-12

**Authors:** Bharat Vaswani, Palanki S Dattatreya, Hanmant Barkate, Sagar B Bhagat, Saiprasad Patil, Amit Y Jadhav

**Affiliations:** 1 Medical Oncology, Yashoda Cancer Institute, Secunderabad, IND; 2 Medical Oncology, Renova Soumya Cancer Hospital, Secunderabad, IND; 3 Global Medical Affairs, Glenmark Pharmaceuticals Limited, Mumbai, IND

**Keywords:** netupitant and palonosetron, patient risk factors, mec, hec, cinv

## Abstract

Background

Female gender, young age, first chemotherapy cycle, and low alcohol intake have all been linked to an increased risk of nausea and vomiting from chemotherapy. We intended to see if netupitant and palonosetron (NEPA) could prevent chemotherapy-induced nausea and vomiting (CINV) in patients with risk factors such as age, gender, chemotherapy cycle number, and alcohol consumption history.

Methods

In this retrospective study, chemotherapy-naïve patients who were prescribed netupitant (300 mg) and palonosetron (0.50 mg) (NEPA) before the first cycle of chemotherapy were analyzed for overall, acute, and delayed phases of complete response (CR), complete protection (CP), and control.

Results

In the acute phase (AP), delayed phase (DP), and overall phase (OP), complete response was 88.23%, 86.27%, and 86.27%, respectively; complete protection was 80.39%, 78.43%, and 76.47%, respectively; and complete control was 76.47%, 72.54%, and 70.58%, respectively, in the whole population (i.e., 51 patients). Complete response, protection, and control in the overall phase were achieved by 86.27%, 72.72%, and 68.18% of patients who received the highly emetogenic chemotherapy (HEC) regimen (i.e., 44 patients), respectively.

Conclusion

NEPA provided a consistent magnitude of benefit for patients who are at high risk, receiving HEC and moderately emetogenic chemotherapy (MEC), and having good control in the acute, delayed, and overall phases of CINV.

## Introduction

Chemotherapy-induced nausea and vomiting (CINV) is one of the most common and undesirable side effects of chemotherapy that not only affects the patient’s quality of life but also leads to decreased compliance with the treatment, affecting the treatment outcome [1]. Because chemotherapy remains the cornerstone therapy in the treatment of various cancers, CINV poses a significant challenge to the treating oncologist. Despite the use of antiemetic therapy as recommended by guidelines, response and adherence remain poor, and nausea affects at least half of patients with cancer [2].

There are two major factors contributing to the persistence of nausea and vomiting following chemotherapy. These factors primarily encompass underestimation of the emetogenic potential of the commonly used chemotherapy and underestimation of the patient-related risk factors associated with CINV. Aside from the National Comprehensive Cancer Network (NCCN), no other guidelines advocate for taking patients’ risk factors into account when selecting chemotherapy and providing effective antiemetic prophylaxis [3-6]. As per Indian expert consensus on CINV, a multifaceted approach including patient risk factors ensures consistent CINV prevention [7]. Various physiological, demographic, and external factors are linked to and increase the risk of CINV in patients [1]. As a result, the best antiemetic prophylaxis should be based on the emetic risk of therapy as well as patient-related factors. Knowledge of patient-related risk factors for CINV prior to the administration of highly emetogenic chemotherapy (HEC)/moderately emetogenic chemotherapy (MEC) may allow for planning an optimal antiemetic regime, which will help in improving overall outcomes.

CINV involves the central pathway of substance P and the peripheral pathway of serotonin, which targets neurokinin 1 (NK-1) and 5-hydroxytryptamine (5HT-3) receptors, respectively [1]. Also, patients have poor compliance and adherence to cancer medications due to CINV. So, to increase compliance and adherence and to target both causative pathways of CINV, the fixed-dose combination of netupitant and palonosetron (NEPA) is approved by leading regulatory authorities such as the US Food and Drug Administration (FDA), European Medicines Agency (EMA), and Drugs Controller General of India (DCGI). NEPA is a fixed combination of the highly selective NK-1 receptor antagonist (netupitant) and the pharmacologically and clinically distinct 5-HT3 receptor antagonist (palonosetron) [4,5].

Prior studies on patients in India [8,9] and the Western world [10-12] found that NEPA had a good response in controlling acute/delayed and overall nausea and vomiting in patients on highly and moderately emetogenic chemotherapy.

The present subgroup analysis was conducted based on data from 329 chemotherapy-naïve patients who received NEPA for CINV prophylaxis between June 2019 and December 2019 [8]. The analysis aimed to evaluate the effectiveness of NEPA in controlling nausea and vomiting among patients with high-risk factors for CINV.

## Materials and methods

Study design and ethics

This was a subgroup analysis of a multicenter, retrospective, observational study that was carried out at two centers in India after receiving ethics committee approval. The medical records of all chemotherapy-naïve patients on HEC or MEC regimens who were prescribed NEPA for CINV prophylaxis and had complete data on nausea and vomiting control were retrieved.

Participants

The following patient-related risk factors were considered, and those patients with all the risk factors were considered for analysis: female gender, young age (<50 years), first cycle of chemotherapy, and no/low alcohol intake. The treatment given was NEPA with dexamethasone as per the treating oncologist’s preference.

Outcome measurement

The responses recorded were the presence or absence of vomiting and nausea. The severity of nausea was recorded on a visual analog scale (VAS) (10 cm). Nausea grading < 2.5 cm was considered as no significant nausea (NSN). The following endpoints were used to describe the effectiveness of NEPA in cycle 1: overall complete response (CR-O), defined as no vomiting and no need for rescue medication in the time frame 0-120 hours; complete response during the acute phase (CR-AP), defined as no vomiting and no need for rescue medication in the time frame 0-24 hours; complete response during the delayed phase (CR-DP), defined as no vomiting and no need for rescue medication in the time frame 24-120 hours; overall complete protection (CP-O), defined as no significant nausea (<2.5 cm on VAS), no vomiting, and no use of rescue medication in the time frame 0-120 hours; and overall complete control, defined as no emetic episodes, no rescue therapy, and no nausea (0 cm on VAS) in the time frame 0-120 hours.

## Results

A total of 329 medical records were reviewed, of which 51 patients had all the risk factors for CINV. Among 51 patients, 44 (86.27%) received HEC, while seven (13.72%) received MEC (Table 1). The average age (± standard deviation (SD)) was 40.98 ± 7.58 years. Breast cancer (21%) was the most common diagnosis in this patient population, followed by ovarian cancer (17%).

**Table 1:** Complete response (no emesis and no use of rescue medication) (n = 51)

Population	Acute phase (0-24 hours)	Delayed phase (24-120 hours)	Overall phase (0-120 hours)
Overall	45 (88.23%)	44 (86.27%)	44 (86.27%)
Highly emetogenic chemotherapy	38 (86.36%)	37 (84.09%)	37 (84.09%)
Moderately emetogenic chemotherapy	7 (100%)	7 (100%)	7 (100%)

In the overall phase (OP), 86.27% achieved complete response (CR), with 88.23% achieving CR in the acute phase and 86.27% achieving CR in the delayed phase (DP). Among those who received the HEC regimen, the CR-AP, CR-DP, and CR-OP rates were 86.36%, 84.09%, and 84.09%, respectively, whereas those who received the MEC regimen demonstrated a 100% complete response in all phases of CINV control (Table 1).

Complete protection (CP) was 76.47% in the overall phase, 80.39% in the acute phase, and 78.43% in the delayed phase. Similarly, complete protection was observed in 77.27%, 75%, and 72.72% of patients given HEC regimens for acute, delayed, and overall phases, respectively, with those on MEC demonstrating 100% CP in all phases (Table 2).

**Table 2:** Complete protection (no emesis, no significant nausea, and no use of rescue medication) (n = 51)

Population	Acute phase (0-24 hours)	Delayed phase (24-120 hours)	Overall phase (0-120 hours)
Overall	41 (80.39%)	40 (78.43%)	39 (76.47%)
Highly emetogenic chemotherapy	34 (77.27%)	33 (75%)	32 (72.72%)
Moderately emetogenic chemotherapy	7 (100%)	7 (100%)	7 (100%)

Complete control was observed in 70.58% in the overall phase, 76.47% in the acute phase, and 72.54% in the delayed phase. In comparison, complete control was achieved in 72.72%, 70.45%, and 68.18% of patients on the HEC regimen in the acute, delayed, and overall phases, respectively. In the acute, delayed, and overall phases, those on the MEC regimen had a complete control rate of 100%, 85.71%, and 85.71%, respectively (Table 3).

**Table 3:** Complete control (no emesis, no nausea, and no use of rescue medication) (n = 51)

Population	Acute phase (0-24 hours)	Delayed phase (24-120 hours)	Overall phase (0-120 hours)
Overall	39 (76.47%)	37 (72.54%)	36 (70.58%)
Highly emetogenic chemotherapy	32 (72.72%)	31 (70.45%)	30 (68.18%)
Moderately emetogenic chemotherapy	7 (100%)	6 (85.71%)	6 (85.71%)

## Discussion

The present subgroup analysis is likely the first of its kind from India to include patient-related multiple risk factors in analyzing CINV control in patients on HEC/MEC regimens, especially when treated with NEPA.

According to a systemic review of 32 studies [3], females have a 2.79 times higher risk of contracting CINV than males (p = 0.04). This was also supported by a study [13] where CINV odds increased with the female gender (odds ratio (OR) = 3.087). In addition, studies have shown being younger increases the risk of CINV in the near future, which is endorsed by a systematic review stating that younger age patients have a 2.59 times higher risk for CINV [3]. Another factor that affects CINV risk is no or low alcohol intake, and based on studies, there was a 1.94-fold increased risk of CINV in these patients [13,14].

In addition to the aforementioned factors, the chemotherapy cycle number (first cycle) is reported to be associated with a higher risk of CINV than the patients receiving later cycles [3]. Another registry found that female gender, age (<50 years), low alcohol intake, and first chemotherapy cycle all increased the risk of CINV by 1.40, 0.99, 1.03, and 6.46 times, respectively [15].

Complete response in our study was 86.27% in the overall phase, 88.23% in the acute phase, and 86.27% in the delayed phase. Complete protection and complete control in the overall phase were 76.47% and 70.58%, respectively. There have been few studies that evaluated CR in females and those of younger age. A subgroup analysis of a NEPA study by Hesketh et al. [16] revealed that the CR rate in females was 82%, while the rate in younger patients (<55 years) was 85%, which was similar to our study results. In a similar study, male patients over the age of 55 years had a higher CR [16]. Another study found that CINV rates increased with younger age in female patients receiving oxaliplatin (from 17.3% over the age of 70 to 22.8% under the age of 50) [17].

There was no difference in CR when we compared the current subgroup analysis complete response to the primary study population (86.21% versus 85.41%) [8]. The effect of aprepitant-palonosetron-dexamethasone on high-risk CINV patients of the female gender, young age (<50 years), little or no alcohol intake, and first chemotherapy cycle showed CR of 87%, 92.7%, and 88.6% in overall, acute, and delayed phases, respectively, which was consistent with our study [18]. Another study found that the aprepitant-ondansetron-dexamethasone had a CR of 68% in patients with high-risk factors (such as female gender, age (<65 years), and little or no alcohol intake) in the overall phase, which was lower than the current study [19]. When compared to a retrospective study of olanzapine-dexamethasone-tropisetron in high-risk patients (female gender, young age (<55 years), low or no alcohol intake), the CR and CP rates in the overall phase were 70% and 62.5%, respectively, indicating that controlling CINV was more difficult in this subset of patients with high-risk factors [20]. In another pooled analysis of three trials of palonosetron-dexamethasone in high-risk patients (female gender, non-habitual alcohol intake, age <55 years, and chemotherapy-naïve status) the CR rate in acute and delayed phases was 78.8% and 57.3%, respectively, which was significantly lower compared to the present study [13].

Because CINV guidelines are the majority based on chemotherapy’s emetic risk, this study’s findings could help oncologists take patient-related risk factors into account to plan a tailored antiemetic regimen. Hence, this intriguing concept can be utilized in the future to manage CINV risk assessments [21]. A small sample size, especially for the MEC, and the retrospective nature of the study limit the study’s validity.

## Conclusions

The current subgroup analysis demonstrated that NEPA is an effective treatment prophylaxis option even for patients with high CINV risk factors (female gender, young age (<50 years), first cycle of chemotherapy, and no/low alcohol intake) who received HEC/MEC chemotherapy regimens.

## References

[1] Janelsins MC, Tejani MA, Kamen C, Peoples AR, Mustian KM, Morrow GR (2013). Current pharmacotherapy for chemotherapy-induced nausea and vomiting in cancer patients. Expert Opin Pharmacother.

[2] Tamura K, Aiba K, Saeki T (2015). Testing the effectiveness of antiemetic guidelines: results of a prospective registry by the CINV Study Group of Japan. Int J Clin Oncol.

[3] Mosa AS, Hossain AM, Lavoie BJ, Yoo I (2020). Patient-related risk factors for chemotherapy-induced nausea and vomiting: a systematic review. Front Pharmacol.

[4] (2021). National Comprehensive Cancer Network (NCCN): NCCN clinical practice guidelines in Antiemetic 2021. https://www.nccn.org/professionals/physician_gls.

[5] Hesketh PJ, Kris MG, Basch E (2020). Antiemetics: ASCO guideline update. J Clin Oncol.

[6] Roila F, Molassiotis A, Herrstedt J (2016). 2016 MASCC and ESMO guideline update for the prevention of chemotherapy- and radiotherapy-induced nausea and vomiting and of nausea and vomiting in advanced cancer patients. Ann Oncol.

[7] Vaid AK, Gupta S, Doval DC (2020). Expert consensus on effective management of chemotherapy-induced nausea and vomiting: an Indian perspective. Front Oncol.

[8] Vaswani B, Dattatreya PS, Bhagat S, Patil S, Barkate H (2021). The effectiveness of NEPA in the prevention of chemotherapy-induced nausea vomiting among chemo naive patients in an Indian setting. BMC Cancer.

[9] Vaswani B, Bhagat S, Patil S, Barkate H (2020). Effectiveness of a novel, fixed dose combination of netupitant and palonosetron in prevention of chemotherapy induced nausea and vomiting: a real-life study from India. World J Clin Oncol.

[10] Hesketh PJ, Rossi G, Rizzi G (2014). Efficacy and safety of NEPA, an oral combination of netupitant and palonosetron, for prevention of chemotherapy-induced nausea and vomiting following highly emetogenic chemotherapy: a randomized dose-ranging pivotal study. Ann Oncol.

[11] Aapro M, Rugo H, Rossi G (2014). A randomized phase III study evaluating the efficacy and safety of NEPA, a fixed-dose combination of netupitant and palonosetron, for prevention of chemotherapy-induced nausea and vomiting following moderately emetogenic chemotherapy. Ann Oncol.

[12] Gralla RJ, Bosnjak SM, Hontsa A (2014). A phase III study evaluating the safety and efficacy of NEPA, a fixed-dose combination of netupitant and palonosetron, for prevention of chemotherapy-induced nausea and vomiting over repeated cycles of chemotherapy. Ann Oncol.

[13] Sekine I, Segawa Y, Kubota K, Saeki T (2013). Risk factors of chemotherapy-induced nausea and vomiting: index for personalized antiemetic prophylaxis. Cancer Sci.

[14] Uomori T, Horimoto Y, Mogushi K, Matsuoka J, Saito M (2017). Relationship between alcohol metabolism and chemotherapy-induced emetic events in breast cancer patients. Breast Cancer.

[15] Molassiotis A, Lee PH, Burke TA, Dicato M, Gascon P, Roila F, Aapro M (2016). Anticipatory nausea, risk factors, and its impact on chemotherapy-induced nausea and vomiting: results from the Pan European Emesis Registry study. J Pain Symptom Manage.

[16] Hesketh PJ, Aapro M, Jordan K, Schwartzberg L, Bosnjak S, Rugo H (2015). A review of NEPA, a novel fixed antiemetic combination with the potential for enhancing guideline adherence and improving control of chemotherapy-induced nausea and vomiting. Biomed Res Int.

[17] Binder G, Saunders WB (2018). Chemotherapy-induced nausea and vomiting (CINV) - incidence by age and sex among patients receiving oxaliplatin. Value Health.

[18] Wang DS, Hu MT, Wang ZQ (2021). Effect of aprepitant for the prevention of chemotherapy-induced nausea and vomiting in women: a randomized clinical trial. JAMA Netw Open.

[19] Hesketh PJ, Aapro M, Street JC, Carides AD (2010). Evaluation of risk factors predictive of nausea and vomiting with current standard-of-care antiemetic treatment: analysis of two phase III trials of aprepitant in patients receiving cisplatin-based chemotherapy. Support Care Cancer.

[20] Wu X, Wu J, Tong G (2020). Efficacy of olanzapine-triple antiemetic regimen in patients with gastrointestinal tumor and high risk of chemotherapy-induced nausea and vomiting receiving moderately emetogenic chemotherapy: a retrospective study. Cancer Manag Res.

[21] Dranitsaris G, Molassiotis A, Clemons M (2017). The development of a prediction tool to identify cancer patients at high risk for chemotherapy-induced nausea and vomiting. Ann Oncol.

